# 17. Inclusion body myositis associated with SLE

**DOI:** 10.1093/rap/rky033.009

**Published:** 2018-09-20

**Authors:** Catrin M Jones, Ceril Rhys-Dillon

**Affiliations:** Rheumatology, Cwm Taf UHB, Llantrisant, UNITED KINGDOM

**Introduction:** This case report describes a 67 year old female patient with SLE who was diagnosed with inclusion body myositis after extensive investigation for symptoms of progressive muscle weakness after initial treatment for presumed SLE-associated myositis.

**Case description:** Initially the patient presented with symptoms of flitting arthralgia, myalgia, mouth ulcers and sicca symptoms. Blood results showed intermittent leucopenia, positive ANA (titre 1: 1600), raised dsDNA 59.1 and low C4 levels 0.12. ENA was negative and creatine kinase levels within normal range. Background of a duodenal GIST (gastrointenstinal stromal tumour) with liver metastases with surgical resection in 2012 and ongoing treatment with tyrosine kinase inhibitor (masitinib) as part of a clinical trial. Treatment with hydroxychloroquine 200 mg bd and a reducing course of oral prednisolone was commenced. Initial response to treatment was favourable with no features of active SLE on subsequent clinic reviews. November 2016 saw worsening muscle weakness with difficulty to from sit to stand and climbing stairs. Evidence of proximal weakness, most severe in hip flexors (power 4/5), particularly in the left leg with preservation of distal power. Blood tests showed a mildly elevated creatine kinase 256, however normal inflammatory markers. MRI imaging of thighs showed subtle asymmetrical signal in the adductor longus muscles and left biceps femoris muscle. MRI Spine showed no cord compression nor metastatic spinal involvement. CT imaging showed no progression of malignancy. EMG showed low amplitude short duration units in keeping with a myopathy but no evidence of myositis. The muscle biopsies from the right medial thigh and left posterior thigh showed patchy vacuolated fibres throughout the muscle fascicles but no inflammation. Lamellated inclusions were observed within many myofibres in association with autophagic vacuoles containing degenerate material. Hydroxychloroquine was stopped due to the possibility of a drug induced vacuolar myopathy. The patient was referred for a neuromuscular opinion at the nearest tertiary centre. The muscle weakness persisted despite withdrawal of hydroxychloroquine. SLE treatment was challenging once hydroxychloroquine was stopped. Masitinib treatment posed a risk of neutropenia, therefore treatment with mycophenolate or azathioprine was difficult. A further complication was a prolonged hospital admission with a Klebsiella cavitating pneumonia in 2017. During this admission patient developed profound thrombocytopaenia (platelets 15) and also had low IgG 4.50. Initially prednisolone was increased to 1 mg/kg. She was later treated with iv immunoglobulins 2g/kg in October 2017 on the basis of presumed SLE-mediated myositis in addition to ongoing thrombocytopaenia and hypogammaglobinaemia. This did result in a documented improvement to graded muscle power, particularly in the legs, but effects were transient. Further course of immunoglobulin 2g/kg due to persistent and recurrent infection was given in March 2018 with similar effect. At neuromuscular specialist review in April 2018 there was further deterioration in mobility and also swallowing difficulties. There was predominant weakness in left wrist flexion and finger flexion and extension Facial muscle and neck flexor weakness were also noted as well a loss of knee jerks. A diagnosis of inclusion body myositis was made based on clinical findings and review of previous investigations. Investigation findings has not been fully conclusive, however the pattern of asymmetrical muscle group involvement, particularly affecting finger flexors associated with loss of knee jerks, swallowing problems and modest creatine kinase elevation were in keeping with this diagnosis. There had also been failure to respond to steroid treatment and evidence of vacuolated muscle fibres from the biopsy. Ongoing management involved referral to the specialist neuromuscular physiotherapist and input from speech and language therapist to address swallowing difficulty.

**Discussion:** Inclusion body myositis is a slowly progressive inflammatory myopathy which is characterised by asymmetrical distal and proximal weakness, particularly affecting the finger and wrist flexors and quadriceps. Inclusion body myositis has been associated with immune mediated conditions, however is usually resistant to treatment with conventional immunotherapies. There have been previous case reports of inclusion body myositis associated with SLE which show similarities to our patient. Two case reports described SLE-associated myositis initially responsive to treatment but later developed inclusion body myositis. Failure to respond to immunosuppressive treatment was reported in the majority of the case reports, but response to methotrexate and corticosteroids was observed in one instance. Interestingly, a response to immunoglobulin treatment was also described, as we observed in our case. Certainly the symptoms and signs observed in our case initially had been suggestive of SLE-associated myositis at the time when her SLE was active, but when the patient developed progressive symptoms despite treatment her diagnosis was re-evaluated. An interesting red herring in this case was the vacuolar muscle biopsy changes suggestive of a hydroxychloroquine-induced myopathy. This was initially thought to be the cause of the symptoms but the muscle weakness continued to progress despite cessation of the drug.

**Key Learning Points:** This case is a further example of inclusion body myositis in association with SLE. It highlights the diagnostic challenges that rheumatologists can face despite extensive investigations when different conditions co-exist. It is a reminder of the importance re-evaluating a clinical diagnosis when a patient fails to respond to treatment. Clinical assessment of symptoms and signs was essential for diagnosis of inclusion body myositis in this case when the investigations were not fully conclusive.

**Disclosure: C.M. Jones:** None. **C. Rhys-Dillon:** None.

